# Individual differences in autistic traits predict the perception of direct gaze for males, but not for females

**DOI:** 10.1186/2040-2392-5-12

**Published:** 2014-02-12

**Authors:** Daisuke Matsuyoshi, Kana Kuraguchi, Yumiko Tanaka, Seina Uchida, Hiroshi Ashida, Katsumi Watanabe

**Affiliations:** 1 Research Center for Advanced Science and Technology, The University of Tokyo, 4-6-1 Komaba, Meguro, Tokyo 153-8904, Japan; 2 Department of Psychology, Graduate School of Letters, Kyoto University, Yoshida-honmachi, Sakyo, Kyoto 606-8501, Japan; 3 College of Arts and Sciences, The University of Tokyo, 3-8-1 Komaba, Meguro, Tokyo 153-8902, Japan

**Keywords:** Direct gaze, Individual differences, Sex differences, Autistic traits, Autism spectrum disorders (ASD)

## Abstract

Despite the emphasis of autism spectrum disorders as a continuum of atypical social behaviors and the sexual heterogeneity of phenotypic manifestations, whether gaze processing constitutes an autistic endophenotype in both sexes remains unclear. Using the Autism-Spectrum Quotient and a psychophysical approach in a normal population (N = 128), here we demonstrated that individual differences in autistic traits predicted direct-gaze perception for males, but not for females. Our findings suggest that direct-gaze perception may not constitute an autistic endophenotype in both sexes, and highlight the importance of sex differences when considering relationships between autistic traits and behaviors.

## Findings

Individuals with autism spectrum disorders (ASD) exhibit atypical behavior in perceiving others’ eye gaze and eye contact, a crucial factor underlying social communication [1,2]. Their heterogeneity of phenotypic manifestations has led researchers to suggest that autistic traits are extending into the normal population [3,4]. Besides individual heterogeneity, sexual heterogeneity of phenotypes has also been suggested in individuals with ASD [5]. However, whether gaze processing constitutes an autistic endophenotype in both sexes remains unclear. By examining the relationship between performance in perceiving direct gaze and the scores on the Autism-Spectrum Quotient (AQ) [6] in a normal population, we show that individual differences in autistic traits predict performance in direct-gaze perception for males, but not for females.

**Figure 1:**
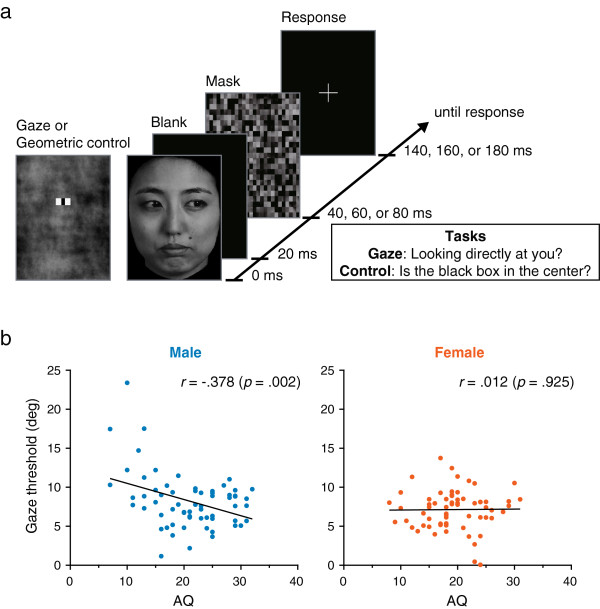
**Experimental paradigm and results. (a)** Schematic presentation of the experimental task. The two tasks were performed in four separate blocks, each containing 144 trials, with the order counterbalanced across participants. **(b)** Correlation between the Autistic-Spectrum Quotient (AQ) score and direct-gaze threshold (estimated angle of 50% ‘direct’ response). A significant correlation was observed in male, but not female participants. A significant correlation between the AQ score and geometric (control) threshold was not observed in both sexes.

Each trial started with the 20-ms presentation of a face or geometric control stimulus (Figure 1a). Face images were looking either directly toward the participant (0°), or left- or right-averted by 10°, 20°, or 30° (see Additional file 1). The control geometric stimuli (a black box embedded in a white rectangle) were adjusted to match the mean apparent sclera-iris ratio of the face images. All stimuli were followed by a 100-ms mask. A variable inter-stimulus interval (ISI) was used to control task difficulty (20, 40, or 60 ms). Following the mask, a fixation cross was presented until a response was obtained.

Participants (64 females, 64 males) were required to indicate whether the eyes were looking directly at them for the gaze task. They were required to indicate whether the black box was in the center of the white rectangle for the geometric control task. Gaze/geometric threshold is defined as the angle at which a 50% direct/center response is achieved, as estimated by fitting a logistic function to each observer’s responses [7] (Additional file 1: Figure S1). A lower threshold indicates higher sensitivity in direct/center perception. Participants also completed the AQ, a questionnaire that assesses autistic traits in normal adults [6].

A significant correlation was observed between the AQ score and gaze threshold in male (*r* = -.378, *P* = .002), but not female participants (*r* = .012, *P* = .925) (Figure 1b). Significant correlations were not found between the AQ score and geometric threshold in male (*r* = -.227, *P* = .071) or female participants (*r* = -.084, *P* = .508) (Additional file 1: Figure S2). A correlation between the AQ score and gaze threshold was greater in male than in female participants (*z* = 2.260, *P* = .024). The correlation between the AQ score and geometric threshold was similar between male and female participants (*z* = .809, *P* = .407). In addition, a partial correlation (controlling for the geometric threshold) between the AQ score and gaze threshold was significant in male (*r* = -.312, *P* = .013), but not in female participants (*r* = .028, *P* = .828), which indicated that the significant correlation was not attributed to low-level discrimination acuity, but was specific to gaze processing. The higher sensitivity in males with high AQ scores may reflect a shift toward typically-developing females [8] and/or higher dependency on low-level visual information in gaze processing [9] (see Additional file 1).

Our results clearly demonstrated that the relationship between gaze perception and autistic traits measured by the AQ may differ between male and female individuals. The perception of direct gaze may constitute an autistic endophenotype in the normal population for males, but not for females. As mechanisms underlying sex differences in the prevalence and phenotypic manifestation of ASD appear to be multifactorial [5,10], at least two possible explanations exist for our findings; these are as follow.

First, female protective factors may underlie sex differences in our study. Recent studies have demonstrated the necessity of a greater genetic load to present autistic behaviors in females than in males [11,12] and have also suggested that some genetic factors related to the female sex protect them against autistic behavior [5,13]. Furthermore, females may learn to effectively mask or camouflage their autistic behaviors through their development and/or experience as female [14]. It is likely that these genetic and non-genetic female protective factors modulate the processing of direct gaze in the general population, rendering the autistic-trait dependency of behavior, which is found in males, invisible in females.

Second, the autistic traits measured by the AQ may not necessarily reflect all autistic behaviors in both sexes and/or may be somewhat male-biased. Consequently, although the AQ does not include an item directly asking about eye gaze, the autistic traits measured by the AQ may be linked with the perception of direct gaze in males only, and not in females. If this is partly valid, our results may imply that gaze processing in females is mediated by, if not totally, distinct mechanisms from males [15].

In conclusion, our results demonstrated the sex-differential correlational-patterns between autistic traits and direct-gaze perception in the general population that may further extend into the extremes of autistic trait distribution (that is, individuals with a clinical diagnosis of autism). More generally, our findings highlight the importance of sex differences when considering relationships between autistic traits and a range of cognitive/behavioral functions, including gaze perception and gaze behavior. Future studies are needed to examine sex differences in order to capture autistic endophenotypes accurately.

## Abbreviations

ASD: autism spectrum disorders; AQ: Autism-Spectrum Quotient; ISI: inter-stimulus interval.

## Competing interests

None of the authors have any biomedical financial interests or potential conflicts of interest.

## Authors’ contributions

DM, KK, HA, and KW designed the study. DM and KK collected stimulus materials. DM, YT, and SU collected the data. DM analyzed the data. All authors contributed to drafting this manuscript. All authors read and approved the final manuscript.

## Supplementary Material

Additional file 1 **Supplemental methods and results.** Supplemental methods presenting detailed methods. Supplemental results and discussion presenting detailed results and related discussion. **Table S1.** Presenting detailed participant characteristics. **Figure S1.** Presenting angle psychometric functions. **Figure S2.** Presenting correlation in the geometric control condition. Click here for file
