# Supplementary material for: Individual differences in autistic traits predict the perception of direct gaze for males, but not for females
Source: Mol Autism. 2014 Feb 12;5:12. doi: 10.1186/2040-2392-5-12 (PMC3926258; doi:10.1186/2040-2392-5-12)
Supplement: Additional file 1 — Supplemental methods and results. Supplemental methods presenting detailed methods. Supplemental results and discussion presenting detailed results and related discussion. Table S1. Presenting detailed participant characteristics. Figure S1. Presenting angle psychometric functions. Figure S2. Presenting correlation in the geometric control condition. [file 2040-2392-5-12-S1.docx]

**Supplemental Material**

**Supplemental Methods**

One hundred and forty-three young Japanese adults participated. Each participant gave written informed consent after being apprised of the procedure, which had been approved by the Committee of Ethics, Research Center for Advanced Science and Technology, The University of Tokyo. The procedure was made in accordance with Declaration of Helsinki.

As the focus of this study was to examine behavior in the normal population, the following exclusion criteria were set. Data from 8 participants were not used because they scored too high (>32) on the AQ [1]. Seven participants were excluded because they did not correctly perform the task (their performance did not reach the 50% level). Data from the remaining 128 participants (64 males, 64 females; mean age: 20.9 years; for details, see Table S1) were analyzed. All participants had normal or corrected-to-normal vision and none reported a history of neurological or developmental disorders.

Participants viewed stimuli on a 17-inch CRT monitor at a distance of 57 cm with their heads on a chin-rest. All stimuli were presented on a black background. Face images were taken from 6 models (3 males, mean age = 24.0 years; 3 females, mean age = 27.0 years) photographed by the authors. The images were gray-scaled and subtended approximately 17 by 24° of the visual angle. The mean brightness was normalized across images. The backgrounds of geometric stimuli were generated by phase-scrambling the corresponding face images. The mask stimulus was generated from mosaic of an averaged image of the face stimuli. Thus, the mean brightness of the gaze, geometric control, and mask stimuli were identical.

For the analysis, responses from left- and right-averted gaze/geometric stimuli were pooled for each absolute angle. Each angle was presented 12 times, except for 0° stimuli, which were presented 24 times, for each of the three ISI conditions for both tasks (576 trials in total). The two tasks were performed in four separate blocks, each containing 144 trials, with the order counterbalanced across participants. The main experiment was preceded by 30 practice trials.

**Table S1. Participant characteristics**

| Mean (±1 SD) | Male | Female | Group comparison |
| --- | --- | --- | --- |
| N | 64 | 64 | - |
| Age | 21.0 (±1.9) | 20.7 (±1.8) | *p* = .418, *d* = .144 |
| Age range | 18-28 | 18-30 | - |
| Handedness (L:R) | 3:61 | 4:60 | *p* = .697, *φ* = .049 |
| AQ | 20.9 (±6.5) | 19.4 (±5.2) | *p* = .145, *d* = .259 |
| Social skill^†^ | 4.2 (±2.7) | 3.8 (±2.2) | *p* = .429, *d* = .140 |
| Attention switching^†^ | 5.0 (±1.8) | 4.6 (±1.9) | *p* = .181, *d* = .238 |
| Attention to detail^†^ | 4.0 (±2.0) | 4.5 (±2.1) | *p* = .209, *d* = .223 |
| Communication^†^ | 3.8 (±2.3) | 3.4 (±1.8) | *p* = .250, *d* = .204 |
| Imagination^†^ | 3.9 (±1.7) | 3.1 (±1.7) | *p* = .009 **, *d* = .471 |

† Sub-scales of the AQ; ** *p* < .01; *d* indicates Cohen’s *d*, *φ* indicates the phi coefficient.

**Supplemental Results and Discussion**

*Psychometric functions*

A repeated-measures ANOVA on participants' direct/center responses with angle (0°, 10°, 20°, 30°) as a within-subject factor and sex (male, female) as a between-subject factor was separately performed with gaze and geometric conditions (Fig. S1). The main effect of the angle was significant in both gaze (*F*(1.770,223.057) = 2528.861, *p* < .0001, $\eta_{p}^{2}$ = .953) and geometric (*F*(1.656,208.617) = 2165.630, *p* < .0001, $\eta_{p}^{2}$ = .945) conditions, which indicated a decrease in direct/center responses with an increase in the angle (*df* = Greenhouse-Geisser corrected). The main effect of sex (*F*(1,126) = 7.095, *p* = .009, $\eta_{p}^{2}$ = .053) and the interaction between the angle and sex (*F*(1.770,223.057) = 3.537, *p* = .036, $\eta_{p}^{2}$ = .027) were significant in the gaze task. Multiple comparisons revealed that males showed greater ‘direct’ responses at angles between 10 and 30° than females (*F*(1,126) values > 5.206, *p* values < .024, $\eta_{p}^{2}$ values > .040), and similar responses at an angle of 0° (*F* < 1, $\eta_{p}^{2}$ = .002) in the gaze task. Neither the main effect of sex (*F*(1,126) = 1.158, *p* = .284, $\eta_{p}^{2}$ = .009) nor the interaction between the angle and sex (*F* < 1, $\eta_{p}^{2}$ = .004) was significant in the geometric task.

The sharper psychometric function of females than males under the gaze condition was consistent with previous studies that reported the superiority of females in gaze processing tasks [2, 3]. These results may stem from the relatively higher levels of social ability in females [e.g., empathizing, see 4].

*Correlation between the AQ score and magnitude of the maximum ‘direct’ response*

One may argue that a significant correlation may exist between the AQ score and magnitude of the maximum ‘direct’ response at an angle of 0° because individuals with high AQ scores may feel less ‘directly stared at’ from a direct-gaze face and vice versa. However, a significant correlation was not observed between the AQ score and response at an angle of 0° (males, *r* = .095, *p* = .458; females, *r* = -.124, *p* = .328), which indicated that the effect of autistic traits is confined to the gaze threshold rather than the magnitude of ‘direct’ response.

*Males with high AQ*

Although males with high AQ scores had a smaller gaze threshold than those with low AQ scores, their performances were almost similar to those of females (Fig. 1b), which suggests that the higher the autistic traits, the more the males exhibited female-like behaviors. In support of this hypothesis, a recent study demonstrated that, in terms of neuroanatomical volume differences, males with ASD exhibited a shift toward typically-developing females [5, 6]. Although its causal effect on behavior remains to be established, it appears reasonable to assume that sex differences in neural substrates affect sex differences in the phenotypic manifestation of ASD. As a result of this effect, males with high AQ scores may exhibit direct-gaze processing performance similar to that of females.

Furthermore, these results are also consistent with the finding that individuals with autism are more likely to depend on local features in gaze processing than individuals without autism [7, 8]. The higher dependency on low-level visual information in gaze processing may lead to a lower direct gaze threshold in males with high AQ scores [see also 9, 10]. Further research is needed to address this issue.

*Detailed statistical analyses on the correlation in the gaze condition*

We conducted 4 types of analysis in addition to that reported in the main text for the following purposes: to confirm the individual contribution of the gaze and geometric processing, and the interaction effect between the gaze and geometric processing (using a multiple regression model), to directly compare differences between males and females (using Fisher’s z-transformation), to assess correlations with a distribution-free, non-parametric test (using Spearman’s rank-order correlation coefficients), and to assess the influences of an outlier.

In summary, all analyses supported the main finding of our study. Significant correlations were found between the AQ scores and gaze threshold in males, but not in females, while correlations between the AQ scores and geometric threshold were not significant in either sex. However, the relationship between the AQ scores and gaze threshold in males was attenuated by a contribution from the interaction between the gaze and geometric processing. Given that individuals with autism were likely to use low-level featural, not configural, information to detect a direct gaze [8], male individuals may depend more on low-level visual information to process a direct gaze than females. Furthermore, the resemblance between the shape of the eye gaze and geometric stimuli used in our study may render male participants to process geometric stimuli similar to the eye gaze. Gaze-like objects or configurations have been shown to trigger responses that are almost identical to the human gaze [11, 12]. Indeed, we found a significant correlation between the gaze and geometric threshold in male (*r* = .516, *p* < .0001), but not in female participants (*r* = .183, *p* = .148). Thus, although geometric processing may play a limited role, it does not fully explain the significant correlation between the AQ score and gaze threshold in males.

We used a multiple regression model with AQ scores as a dependent variable and assessed the interaction term between the gaze and geometric threshold. This analysis not only confirmed the significant effect of the gaze threshold (*β* = -.290, *t* = 2.098, *p* = .040), but also revealed a significant interaction effect on the AQ scores in males (*β* = -.329, *t* = 2.079, *p* = .042). The effect of the geometric threshold was not significant in males (*β* = .142, *t* =.877, *p* = .384). No significant effects were observed in females for the gaze (*β* = .028, *t* =.215, *p* = .831), geometric threshold (*β* = -.090, *t* = .683, *p* = .497), or the interaction term (*β* = -.002, *t* = .015, *p* = .988). These results suggest the existence of the combined effects of gaze and geometric processing in males, but not in females.

In addition, we directly compared correlation coefficients between males and females. We used Fisher’s z-transformation and tested the significance of differences in correlations between males and females. A correlation between the AQ score and gaze threshold was greater in male than in female participants (*z* = 2.260, *p* = .024). The correlation between the AQ score and geometric threshold was similar between male and female participants (*z* = .809, *p* = .407). A partial correlation (controlling for the geometric threshold) between the AQ score and gaze threshold was marginally greater in male than in female participants (*z* = 1.938, *p* = .052).

We also assessed correlations with a distribution-free, non-parametric test (Spearman’s rank-order correlation coefficient). A significant correlation was observed between the AQ score and gaze threshold in male (*ρ* = -.262, *p* = .037), but not in female participants (*ρ* = .092, *p* = .468). Significant correlations were not found between the AQ score and geometric threshold in male (*ρ* = -.130, *p* = .306) or female participants (*ρ* = -.087, *p* = .497). A partial correlation (controlling for the geometric threshold) between the AQ score and gaze threshold was marginally significant in male (*ρ* = -.230, *p* = .069), but not in female participants (*ρ* = .113, *p* = .377).

Finally, we assessed the influences of an outlier. We excluded data from a participant whose gaze threshold was approximately 25 in the gaze condition, and examined the relationship between the AQ score and gaze/geometric threshold in males. A significant correlation was observed between the AQ score and gaze threshold (*r* = -.320, *p* = .011), and marginally significant correlation was found between the AQ score and geometric threshold was (*r* = -.224, *p* = .077). A partial correlation (controlling for the geometric threshold) between the AQ score and gaze threshold was marginally significant (*r* = -.238, *p* = .063).

These results not only revealed a significant correlation between the AQ score and gaze threshold in males, but not in females, but also suggest that low-level geometric discrimination acuity may contribute to the correlation observed in males to some degree. However, this low-level visual processing is not sufficient to explain the significant correlation between the AQ score and gaze threshold in males.


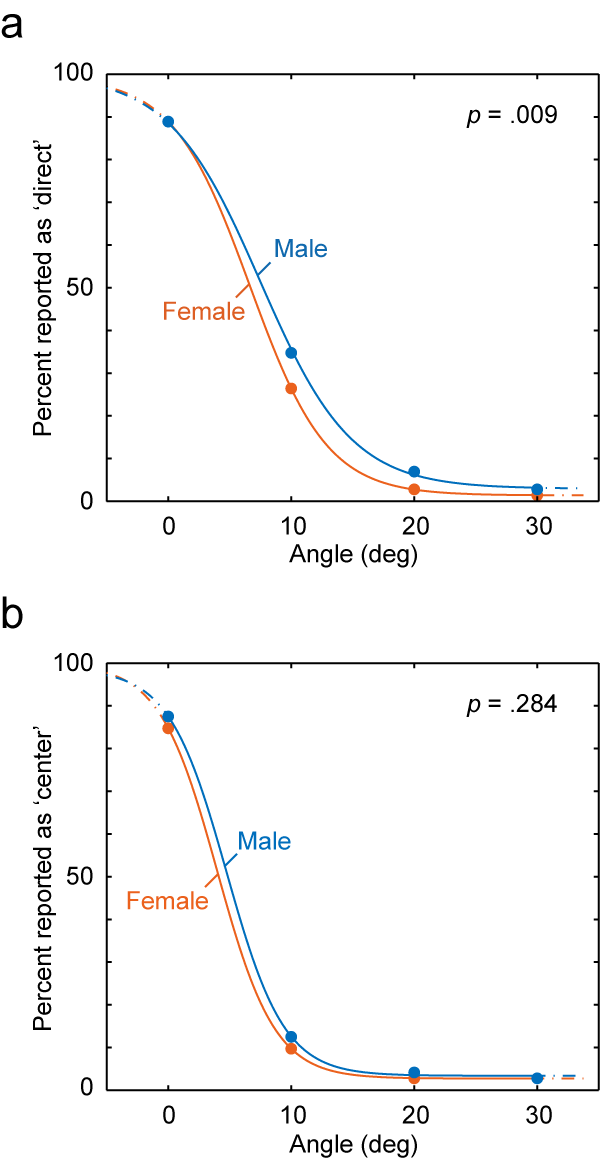


**Fig. S1. Angle Psychometric Functions.** (a) Percentage of responses in which participants reported as ‘direct’ under the gaze condition, plotted as a function of gaze angle. (b) Percentage of responses in which participants reported as ‘center’ under the geometric control condition. *P* values indicate group differences between males and females.


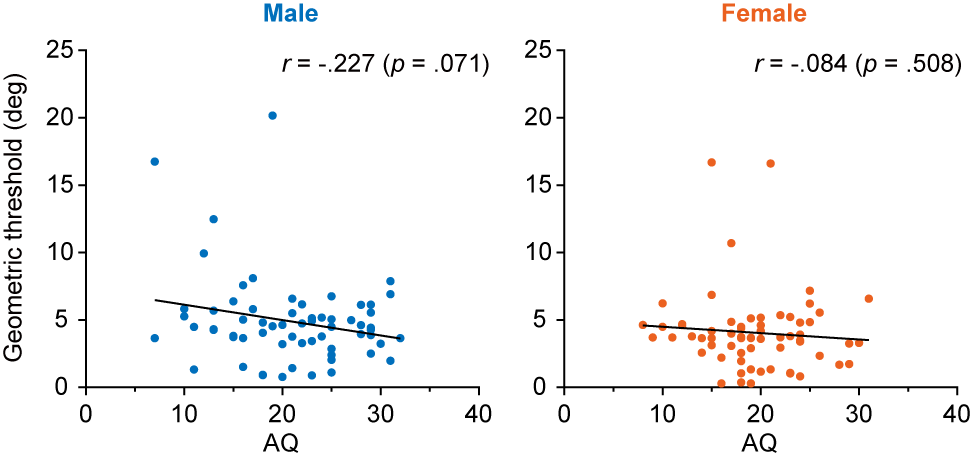


**Fig. S2. Correlation in the Geometric Control Condition.** Correlation between the AQ score and geometric threshold (angle of 50% reported as ‘center’, which was estimated from the psychometric function) for males and females. A significant correlation was not observed in both sexes. Note that, controlling for this geometric threshold, the partial correlation between the AQ score and direct-gaze threshold was significant.

**REFERENCES**

1. Baron-Cohen S, Wheelwright S, Skinner R, Martin J, Clubley E: **The autism-spectrum quotient (AQ): Evidence from Asperger syndrome/high-functioning autism, males and females, scientists and mathematicians.** *J Autism Dev Disord* 2001, **31:**5-17.

2. Goodman LR, Phelan HL, Johnson SA: **Sex differences for the recognition of direct versus averted gaze faces.** *Memory* 2012, **20:**199-209.

3. Bayliss AP, di Pellegrino G, Tipper SP: **Sex differences in eye gaze and symbolic cueing of attention.** *Q J Exp Psychol* 2005, **58A:**631-650.

4. Baron-Cohen S: **The extreme male brain theory of autism.** *Trends Cogn Sci* 2002, **6:**248-254.

5. Lai M-C, Lombardo MV, Suckling J, Ruigrok ANV, Chakrabarti B, Ecker C, Deoni SCL, Craig MC, Murphy DGM, Bullmore ET, et al: **Biological sex affects the neurobiology of autism.** *Brain* 2013, **136:**2799-2815.

6. Lai M-C, Lombardo MV, Chakrabarti B, Bullmore ET, Baron-Cohen S, Consortium MA, Suckling J: **Sex-differential fractal complexity of resting-state brain oscillations in autism.** In *International Meeting for Autism Research*. *2 May 2013; Donostia/San Sebastián, Spain*. 2013.

7. Senju A, Hasegawa T, Tojo Y: **Does perceived direct gaze boost detection in adults and children with and without autism? The stare-in-the-crowd effect revisited.** *Vis Cogn* 2005, **12:**1474-1496.

8. Senju A, Kikuchi Y, Hasegawa T, Tojo Y, Osanai H: **Is anyone looking at me? Direct gaze detection in children with and without autism.** *Brain Cogn* 2008, **67:**127-139.

9. Ashwin E, Ashwin C, Rhydderch D, Howells J, Baron-Cohen S: **Eagle-eyed visual acuity: an experimental investigation of enhanced perception in autism.** *Biol Psychiatry* 2009, **65:**17-21.

10. Mottron L, Dawson M, Soulières I, Hubert B, Burack J: **Enhanced perceptual functioning in autism: an update, and eight principles of autistic perception.** *J Autism Dev Disord* 2006, **36:**27-43.

11. Frischen A, Bayliss AP, Tipper SP: **Gaze cueing of attention: Visual attention, social cognition, and individual differences.** *Psychol Bull* 2007, **133:**694-724.

12. von Grünau M, Anston C: **The detection of gaze direction: A stare-in-the-crowd effect.** *Perception* 1995, **24:**1297-1313.
